# Assessing the RP-LC-MS-Based Metabolic Profile of *Hass* Avocados Marketed in Europe from Different Geographical Origins (Peru, Chile, and Spain) over the Whole Season

**DOI:** 10.3390/plants12163004

**Published:** 2023-08-20

**Authors:** Irene Serrano-García, Joel Domínguez-García, Elena Hurtado-Fernández, José Jorge González-Fernández, José Ignacio Hormaza, María Gemma Beiro-Valenzuela, Romina Monasterio, Romina Pedreschi, Lucía Olmo-García, Alegría Carrasco-Pancorbo

**Affiliations:** 1 Department of Analytical Chemistry, Faculty of Sciences, University of Granada, Ave. Fuentenueva S/N, 18071 Granada, Spain; iserrano@ugr.es (I.S.-G.); jdogar99@correo.ugr.es (J.D.-G.); gemabv@ugr.es (M.G.B.-V.); rmonasterio@ugr.es (R.M.); alegriac@ugr.es (A.C.-P.); 2 Department of Biological and Health Sciences, Faculty of Health Sciences, University of Loyola, Campus Sevilla, Avda. de las Universidades S/N, 41704 Dos Hermanas, Spain; emhurtado@uloyola.es; 3 Institute for Mediterranean and Subtropical Horticulture (IHSM La Mayora-UMA-CSIC), 29750 Algarrobo-Costa, Spain; jorgegonzalez-fernandez@eelm.csic.es (J.J.G.-F.); ihormaza@eelm.csic.es (J.I.H.); 4 Instituto de Biología Agrícola de Mendoza (IBAM), UNCuyo-CONICET, Facultad de Ciencias Agrarias, Chacras de Coria, Mendoza 5505, Argentina; 5 Escuela de Agronomía, Facultad de Ciencias Agronómicas y de los Alimentos, Pontificia Universidad Católica de Valparaíso, Calle San Francisco S/N, La Palma, Quillota 2260000, Chile; romina.pedreschi@pucv.cl; 6 Millennium Institute Center for Genome Regulation (CRG), Santiago 8331150, Chile

**Keywords:** *Hass*, avocado mesocarp, geographical origin, phenolic compounds, pantothenic acid, abscisic acid, amino acids, nucleosides

## Abstract

Spain dominates avocado production in Europe, with the *Hass* variety being the most prominent. Despite this, Spanish production satisfies less than 10% of the overall avocado demand in Europe. Consequently, the European avocado market heavily relies on imports from overseas, primarily sourced from Peru and Chile. Herein, a comprehensive characterization of the metabolic profile of *Hass* avocado fruits from Spain, Peru, and Chile, available in the European market throughout the year, was carried out. The determination of relevant substances was performed using high- and low-resolution RP-LC-MS. Remarkable quantitative differences regarding phenolic compounds, amino acids, and nucleosides were observed. Principal component analysis revealed a natural clustering of avocados according to geographical origin. Moreover, a specific metabolic pattern was established for each avocado-producing country using supervised partial least squares discriminant analysis. Spanish fruits exhibited high levels of coumaric acid malonyl-hexose II, coumaric acid hexose II, and ferulic acid hexose II, together with considerably low levels of pantothenic acid and uridine. Chilean avocado fruits presented high concentrations of abscisic acid, uridine, ferulic acid, succinic acid, and tryptophan. Fruits from Peru showed high concentrations of dihydroxybenzoic acid hexose, alongside very low levels of p-coumaric acid, ferulic acid, coumaric acid malonyl-hexose I, and ferulic acid hexose II.

## 1. Introduction

The avocado (*Persea americana* Mill.) is a subtropical evergreen fruit tree crop native to Mesoamerica. Of the at least eight botanical varieties or subspecies usually recognized, three of them, known as horticultural races, present agronomic importance: West Indian, Guatemalan, and Mexican [1,2]. They are sexually intercompatible but exhibit different botanical traits and edaphoclimatic preferences [3]. Most commercial avocado varieties currently grown in subtropical and Mediterranean climates are inter-racial Mexican × Guatemalan hybrids. An example is *Hass,* the most widespread, cultivated, and marketed variety worldwide [4], which originated as a chance seedling in California ninety years ago [5]. *Hass* is also the most important cultivated variety in Spain, with more than 80% of the acreage and continuing to gain share over other varieties that are still grown, such as *Fuerte* or *Bacon*. *Hass* avocado presents a great environmental plasticity, a long harvesting period, and a hard skin that hides damages and bruises during handling. In addition, it shows a long post-harvest life and good adaptation to pre-ripening, thereby enhancing fruit storage. For consumers, it offers good organoleptic attributes and easy identification of the ideal moment of consumption due to the change in the fruit skin color from green to dark violet/black as the fruit ripens [6]. 

Among the major tropical fruits, avocados have experienced the fastest growth in global output and international trade over the last decades. The predictions for 2030 project that global avocado production will reach 12 Mt, more than three times its level in 2010, and that avocado will become the most traded tropical fruit in international markets. According to the Food and Agriculture Organization of the United Nations, a total of 69 countries distributed around the world produced avocados in 2021 [7]. Mexico ranked first, followed by Colombia, Peru, Indonesia, and the Dominican Republic. Spain is far from the top growing countries, although it is of great importance as the main producer in the Mediterranean area, together with Israel and Morocco. Avocado commercial cultivation started in Spain in the 1950s, but the main expansion took place in the 1970s [8]. Avocado cultivation in Spain is concentrated in the Southern Mediterranean coast (provinces of Malaga and Granada) as well as in the Canary Islands, with recent increasing expansion to the East (Valencian Community) and West (provinces of Huelva and Cadiz) of the country. The production in Spain reached close to 117,000 tons of fruit/year in 2021 [7]. More than 90% of the European Union’s avocado production comes from Spain, although Spanish production, which in the case of *Hass* is concentrated between November and March, only covers about 10% of the total European consumption. Therefore, most of the *Hass* avocado volume marketed in Europe is imported from South America and Africa, with Peru and Chile as the main suppliers. Peru dominates the summer supply, whereas Chilean avocados are traditionally covering the gap between Peruvian and Spanish productions in autumn. Thus, having in the market a single variety (*Hass*) from very different geographical and edaphoclimatic origins results in a lack of homogeneity of the avocados available in Europe.

The avocado fruit is principally composed of monounsaturated and polyunsaturated fatty acids, but also carbohydrates, proteins, and fiber [9,10]. It also contains some relevant vitamins and minerals [11]. The minor fraction of avocado includes mainly phenolic compounds, carotenoids, and terpenoids and has been extensively studied for its biological activity and its relationship with beneficial health effects [9,10,11]. Nevertheless, the compositional profile of the avocado fruit depends on a huge number of factors, such as the variety, climatic conditions, orchard location, or pre- and post-harvest biotic and abiotic stresses [3,12]. All this may lead to the presumption that avocado fruits of the same variety, grown in different countries with different edaphoclimatic conditions and subjected to distinct pre- and post-harvest treatments (depending on whether or not they are exported), will have a different compositional profile. Several works have assessed a correlation between some nutritional components of the avocado and the producing region. Landahl and co-authors and Donetti and Terry determined fatty acids and C_7_ sugars, among other parameters, in *Hass* avocados grown in Chile, Peru, and Spain [13,14]. Both works described that the composition of fatty acids varied significantly according to the geographical origin; Donetti and Terry suggested oleic acid as a potential marker to distinguish fruit origin [14]. With the same objective, Tan and collaborators analyzed *Hass* avocado oils from Mexico, Australia, the United States, and New Zealand [15]. Moreover, the impact of orchard altitude and fruit maturity on the fatty acid content of *Hass* avocado fruits from Colombia was evaluated by Carvalho and Velásquez [16]. In the same country, Henao-Rojas and colleagues analyzed physical, chemical, and nutritional parameters associated with the quality of *Hass* avocado in eight localities of the department of Antioquia [17]. Other studies combined the lipid chromatographic fingerprint with powerful chemometrics tools to classify avocado samples according to their geographical origin and botanical variety [18,19]. In addition, Muñoz and co-authors approached for the first time the evaluation of the isotopic composition of five light bio-elements (C, N, S, H, and O), together with the mineral content, in avocado samples from eight producing regions [20]. In contrast, metabolomic approaches focused on the determination of other types of compounds (phenolic substances, organic acids, amino acids, etc.) have been little exploited for this purpose. They have previously been used for other goals, such as the comprehensive characterization of avocado tissues [21,22,23,24,25], varietal discrimination [26,27], and assessment of the impact of fruit transport (short- and long-distance) on certain primary and secondary metabolites [28,29].

To the best of our knowledge, no work has been published that evaluates the metabolic profile of *Hass* avocados from different origins in the same market over a whole year, including domestic production and imported fruit. Therefore, the aim of the present study was multiple: (1) to provide a detailed analysis of the metabolic profile (RP-LC-MS-based) of *Hass* avocados marketed in the European market from three different origins, and (2) to find some compounds that could act as potential origin markers, grouping *Hass* avocado fruits according to their geographical provenance (Spain, Peru, and Chile).

## 2. Results and Discussion

### 2.1. Qualitative Characterization of the Metabolic Profile of Avocado cv. Hass by LC-MS

An in-depth qualitative characterization of the metabolic profiles of *Hass* fruits from Spain, Chile, and Peru was carried out using LC-QTOF MS (MS and MS/MS data). Among all the compounds detected, we focused on those with the highest intensity in the chromatographic profiles of the samples from the different geographical origins, selecting 28 substances. Of these, 22 could be identified (at least tentatively), and 5 remained unknown (see Table 1). Table 1 includes the compound annotation, chemical family, retention time (Rt), experimental and theoretical *m/z* of the pseudo-molecular ion, fragments observed in MS/MS analyses, and the calculated molecular formula. Peak identification was achieved by considering accurate MS data, relative retention times, and MS/MS fragmentation patterns. The use of pure standards together with information from previously published reports [21,22,23,24,25,30,31] and several databases (FooDB—www.foodb.ca (accessed on 5 August 2023), MassBank Europe Mass Spectral DataBase—www.massbank.eu (accessed on 5 August 2023), MassBank of North America—www.mona.fiehnlab.ucdavis.edu (accessed on 5 August 2023), etc.) was essential to support the identifications described in the present work. 

Different chemical compounds such as amino acids and derivatives, nucleosides, organic acids, phenolic compounds, vitamins, and phytohormones were identified in the chromatographic profiles. As usual in negative polarity, the predominant ion observed was the [M-H]^-^ (i.e., the pseudo-molecular ion). In elution order, uridine (nucleoside) at *m/z* 243.0610 was the most polar compound monitored and eluted at 2.7 min. Tyrosine with *m/z* 180.0662 (amino acid) and succinic acid with *m/z* 117.0192 (organic acid) were the next to elute, and their identity was corroborated with the pure standard of each. The signal *m/z* 315.0715 at 4.9 min was tentatively assigned to dihydroxybenzoic acid hexose, which has already been reported by different authors [30,32]. The MS/MS analyses showed a fragment with *m/z* 152.9 [M-H-162]^-^, corresponding to the loss of a hexose moiety. The peaks of phenylalanine (amino acid) with *m/z* 164.0720 and pantothenic acid (vitamin) with a predominant MS signal at *m/z* 218.1028 were annotated using their pure standard, whereas the signal with *m/z* 299.0770 at 6.3 min was tentatively assigned to hydroxybenzoic acid hexose [30]. Both hydroxybenzoic acid derivatives (with Rt 4.9 and 6.3 min, respectively) had the same relative elution order as that observed in the study just cited, a fact that reinforces the assignment given. Tryptophan (amino acid), with a main.

MS signal at *m/z* 203.0818, eluted at 6.5 min, followed by the *m/z* 222.0772, which was tentatively assigned as *N*-acetyl-tyrosine. The MS/MS analysis revealed a fragmentation pattern giving a primary fragment of *m/z* 179.9 [M-H-42]^-^, which would be a plausible fragmentation with the suggested identity (breakage by the linkage between the acetyl group and the tyrosine moiety). Moreover, predicted MS/MS spectra coming from the available databases also support the attributed annotation. Chlorogenic acid (phenolic acid) with *m/z* 353.0887 was corroborated using its pure standard. Note that the term ‘chlorogenic acids’ embraces a large group of naturally occurring substances; in this research, we focus on the determination of an outstanding compound of this chemical class, which is assigned the trivial name of chlorogenic acid (CAS number 327-97-9). Coumaric acid hexose isomers I and II (two hydroxycinnamic acid derivatives), with MS signals at *m/z* 325.0934 and 325.0917, respectively, were previously described in avocado mesocarp by Serrano-García and co-authors [28]. In this and other cases, when several isomers of a substance are found, they are indicated with I and II, respectively. Two additional hydroxycinnamic acid derivatives (ferulic acid hexose isomers I and II) were detected at 7.8 and 81 min, respectively, giving the prevailing MS signals with *m/z* 355.1033 and 355.1027, apiece. Both compounds were also previously identified by Campos, Hurtado-Hernandez, López-Cobo, and their respective co-authors [22,30,32]. The signal of epicatechin at *m/z* 289.0718 ([M-H]^-^) was detected at 8.3 min, and another amino acid derivative (*N*-acetyl-leucine) was identified at 8.6 min with *m/z* 172.0974. The coumaric acid-derived compounds previously reported by Serrano-García and collaborators were detected herein, yielding *m/z* signals of 367.1021 and 367.1027, respectively [28]. The next compound found, following the chromatographic elution order, was annotated as *N*-acetyl-phenylalanine, bearing in mind its prevalent MS signal (*m/z* 206.0817), the MS/MS experimental data and the MS/MS spectra found in the consulted databases. The fragment observed with *m/z* 163.9 [M-H-42]^-^ would correspond to the release of the phenylalanine moiety. Finally, the identification of *p*-coumaric acid (phenolic acid), ferulic acid (phenolic acid), and abscisic acid (ABA) (phytohormone) with MS signals of *m/z* 163.0395, 193.0498, and 263.1286, respectively, was based on the use of their pure standards. 

Five other unknown compounds with MS signals of *m*/*z* 337.1139, 341.1242, 187.0976, 287.1492, and 171.1020 were detected within the profiles with considerable intensity, but it was not possible to assign an identity to them. We believe, in any case, that this is not a negative aspect of the work and that the complete elucidation of the identity of these substances is beyond the scope of the present contribution.

**Figure 1:**
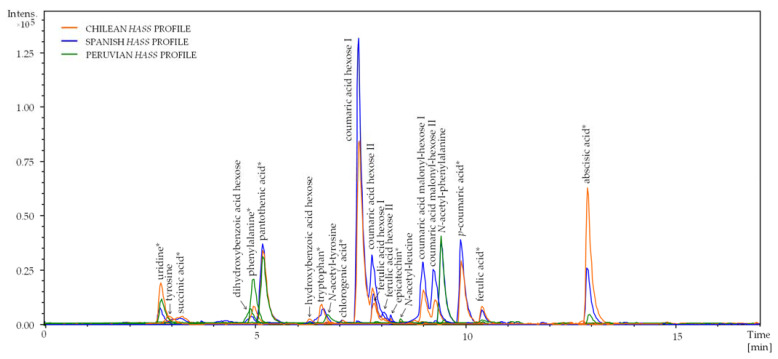
Extracted Ion Chromatograms (EIC) of the most abundant substances within the metabolic profiles obtained from *Hass* avocado extracts from Spain, Chile, and Peru. * Corroborated with the pure standard.

Figure 1 includes examples of the metabolic profiles of avocado extracts from three different geographical origins (Spain, Chile, and Peru), displaying the Extracted Ion Chromatograms of the most abundant substances. The profiles turned out to be quite similar from a qualitative point of view regardless of their origin; however, interesting quantitative differences were observed among origins. They will be discussed in the next sections of this article.

### 2.2. Analytical Parameters of the Method

The analytical performance of the applied method was tested considering linearity, the limits of detection (LODs), the limits of quantification (LOQs), and precision (*intra*- and *inter*-day). The obtained results were satisfactory for every parameter (see Table S1). R^2^ was higher than 0.994 for all the calibration curves, which shows the excellent linearity in each established range and, therefore, the reliability of the quantifications performed. The *intra*-day repeatability ranged between 7.15 and 9.30% (CV values for succinic and ferulic acids, respectively), and *inter*-day repeatability varied from 7.83 to 14.63%, for ABA and succinic acid, apiece. These data show the good precision of the method used. LODs and LOQs ranged between 7.7 and 118.7 µg L^−1^ and 23.6 and 395.4 µg L^−1^ for ABA and succinic acid, respectively.

### 2.3. Quantitative LC-MS Results

**Figure 2:**
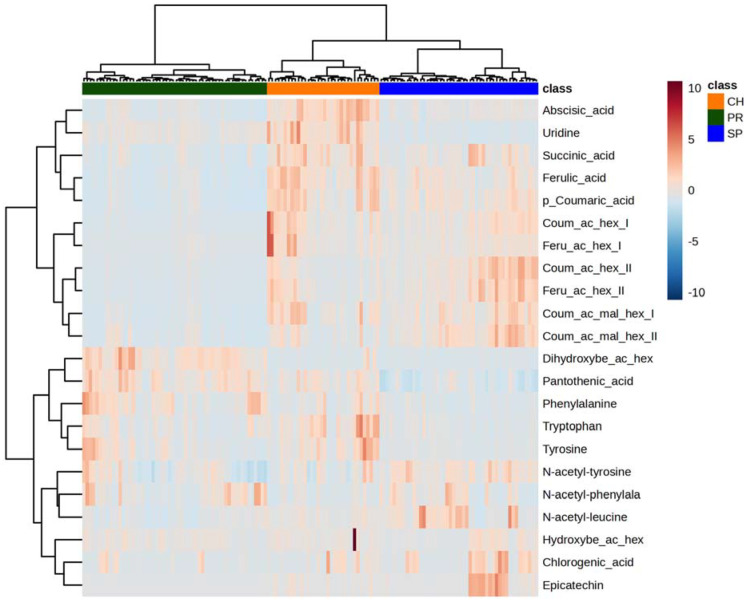
Heat map of the twenty-two compounds quantified for the set of *Hass* avocado samples from Spain, Chile, and Peru.

For quantitative purposes, all avocado extracts considered in this study were analyzed using the LC-ESI-IT MS analytical platform. Initially, the results of each and every sample from the different locations were evaluated in detail, and a heat map considering all samples and compounds was generated (Figure 2). This representation revealed some similarities in the compositional profiles of samples coming from the same geographical origin during the whole sampling period considered in each case. In fact, they were clustered together according to the country of provenance. Nevertheless, as the main aim of this work was to identify the typical compositional patterns of the avocados of each geographical origin, we decided to perform a different type of quantitative data assessment. Therefore, to facilitate the visualization and understanding of the data, the concentrations found for each identified compound were aggregated according to geographical origin to describe what could be considered the “average C18 LC-MS-based metabolic profile” for each country. The results were expressed as mg kg^−1^ of dry weight (DW) together with the associated standard deviation (SD), as shown in Table 2 and in Figure S1. The SD values were considerably high given the design of this study, which covered relatively wide time intervals for sampling (up to 6 months) and a significant number of horticultural replicates (*n* = 5) for each time point. Compounds were classified into six families: amino acids (or related compounds) and nucleosides, flavonoids, organic acids, phytohormones, phenolic acids and related compounds, and vitamins.

Some notable compositional differences were observed among the avocado samples according to their geographical origin. Regarding amino acids and nucleotides, uridine stood out above the others, ranging from 18.68 ± 6.85 mg kg^−1^ DW in Spanish fruits to 96.72 ± 10.14 mg kg^−1^ DW in Chilean avocado samples. Peruvian fruits, in general terms, exhibited higher contents of amino acids and related compounds than Chilean or Spanish fruits, especially for phenylalanine (18.11 ± 7.30 mg kg^−1^ DW) and *N*-acetyl-tyrosine (mean values of 24.25 ± 15.84 mg kg^−1^ DW). Tyrosine content was higher in Chilean (9.18 ± 5.85 mg kg^−1^ DW) than in Spanish (2.62 ± 0.76 mg kg^−1^ DW) fruits, similar to the observations in a previous work [29].

Flavonoids are synthetized by plants in response, among other factors, to microbial infections and external stresses [33]. Epicatechin was the only flavonoid quantified in the present study, and its highest content was found in Spanish fruits (27.82 ± 17.48 mg kg^−1^ DW). This average concentration is similar to previous results for fruits of the same variety reported by Hurtado-Fernández and colleagues and Serrano-García and co-authors [28,34]. High standard deviation values for this compound were observed for all geographical origins. This is probably because fruits from the same origin but harvested at different times of the season were analyzed. Fruits from Peru showed by far the lowest flavonoid content (0.05 ± 0.03 mg kg^−1^ DW).

Furthermore, organic acids are not only involved in important pathways of plant anabolism and catabolism but also play an essential role in the organoleptic properties, quality, microbial stability, and consistency of plant foods [35]. Succinic acid was quantified in the present work, and it had been previously reported in avocado mesocarp [22,23,29,30,32]. The concentration of this organic acid fluctuated from 1295.68 ± 263.90 mg kg^−1^ DW in Chilean avocados to 383.93 ± 139.43 mg kg^−1^ DW in Peruvian avocados. Lower concentrations have been previously reported by Hurtado-Fernández et al. [32], and higher concentrations have been found by Campos and Ramos-Aguilar and their respective collaborators [23,30]. As in the present work, Pedreschi and co-authors reported similar succinic acid contents and found higher amounts of this analyte in Chilean than in Spanish *Hass* avocados [29].

As far as ABA is concerned, the highest average concentrations were found in the Chilean fruits (30.81 ± 7.77 mg Kg^−1^ DW), while the Spanish and Peruvian fruits were more similar (11.22 ± 3.87 mg kg^−1^ DW and 7.89 ± 3.42 mg kg^−1^ DW, respectively). The differences in ABA contents could be associated with the role of this phytohormone during fruit softening and the sensitivity to external stresses and other factors. Pantothenic acid, also called vitamin B5, ranged from 20.15 ± 4.84 mg kg^−1^ DW for Spanish fruits to 35.14 ± 6.13 mg kg^−1^ DW for Peruvian ones. Its mean concentration in Chilean fruits was 30.26 ± 4.04 mg kg^−1^ DW. Similar concentration levels of both compounds (ABA and pantothenic acid) have been previously observed by Serrano-García et al. in *Hass* avocados from Spain [28].

Phenolic acids and related compounds conformed the category with the highest number of compounds. The avocado mesocarp, together with its peel and seed, is an important source of phenolic compounds, and its extracts show valuable antioxidant activities [36]. *p*-Coumaric acid and its derivatives were the most abundant phenolic compounds found in the pulp. This is consistent with what has been observed in a recent study [28]. Avocados from Chile showed the highest levels of *p*-coumaric and ferulic acids, with 75.40 ± 34.52 mg kg^−1^ DW and 13.57 ± 3.56 mg kg^−1^ DW, respectively, although not significantly higher than the contents of avocados from Spain with 52.54 ± 20.80 mg kg^−1^ DW and 8.08 ± 2.46 mg kg^−1^ DW. Peru was undoubtedly the origin with the lowest mean concentration of phenolic compounds. 

As noted above, the compounds structurally related to *p*-coumaric acid were more prominent than those from ferulic acid. Coumaric acid hexose isomer I was greater in fruits from Chile (212.37 ± 30.49 mg kg^−1^ DW), whereas coumaric acid hexose isomer II was higher in avocados from Spain (49.08 ± 33.63 mg kg^−1^ DW). With regard to coumaric acid malonyl-hexoses, isomer I was found at relatively similar concentrations in samples from Chile and Spain (49.52 ± 28.71 mg kg^−1^ DW and 44.70 ± 18.37 mg kg^−1^ DW, respectively), but the contents of isomer II were significantly higher in Spanish avocados, with 51.54 ± 21.28 mg kg^−1^ DW. The two hexoses of ferulic acid showed lower concentrations in all origins. Ferulic acid hexose I was the most prevalent but in no case showed as high amounts as the coumaric acid derivatives. 

For the two hexoses related to hydroxybenzoic acid, the same pattern (among the countries) was observed, both appearing in much higher concentrations in Peruvian avocados. The amount of dihydroxybenzoic acid hexose found in Peru (35.96 ± 11.30 mg kg^−1^ DW) was much higher than in Chile or Spain (9.25 ± 4.21 mg kg^−1^ DW and 6.18 ± 0.94 mg kg^−1^ DW, respectively). Several isomers of hydroxybenzoic acid derivatives have been previously quantified in Spanish and Chilean *Hass* avocados [29]; however, as the results of that study did not specify the pure standard with respect to which the quantification was carried out, it is not possible to make a direct comparison with our concentration values. Nonetheless, Chilean avocados exhibited higher amounts of these isomers (especially of what the authors called isomer 3) than Spanish ones, which was also observed in our study. Chlorogenic acid was the compound with the lowest concentration within this family, and its maximum content was 1.89 ± 1.49 mg kg^−1^ DW (Spain). In a previous work, Hurtado-Fernández and colleagues quantified chlorogenic acid in avocado cv. *Hass,* but it was found only in green unripe fruits [32]. Chlorogenic and neochlorogenic acids are dominant in the avocado peel and seed but not in the pulp, which is consistent with our results [24,25]. 

**Figure 3:**
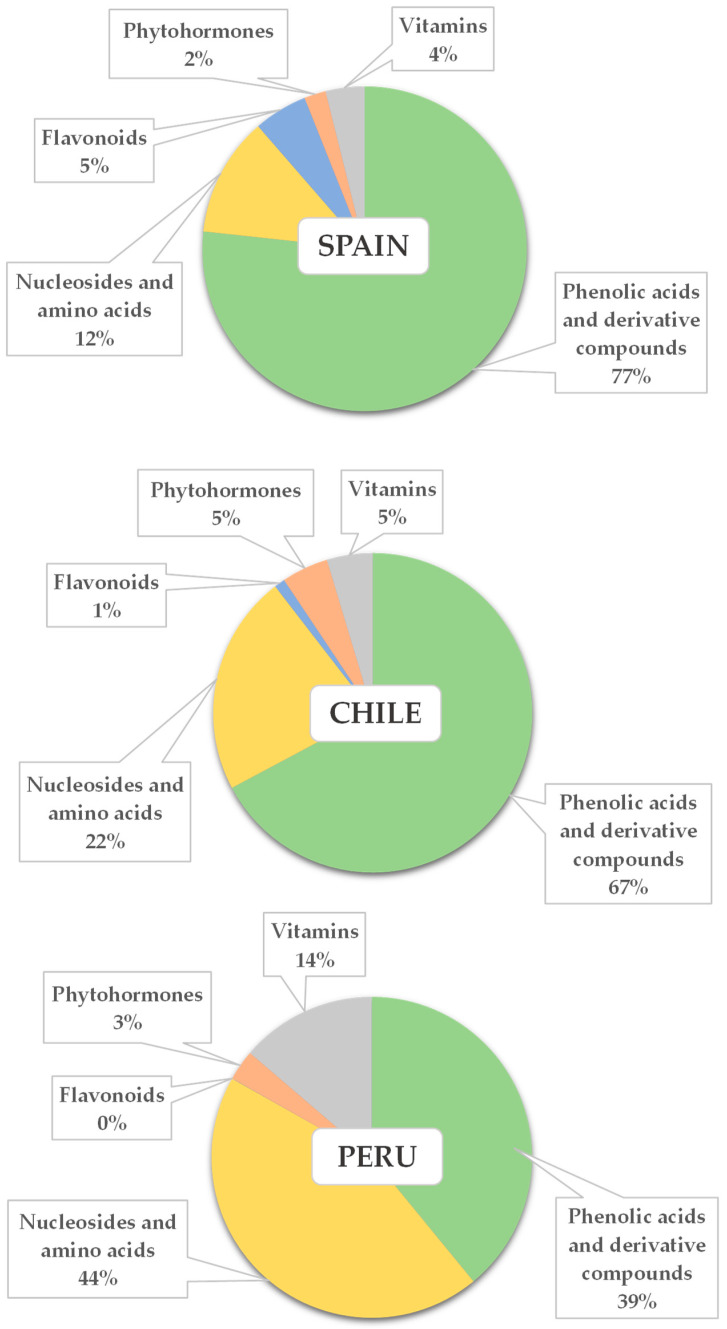
Pie charts of the percentages (%) represented by each chemical family in *Hass* avocados metabolic profiles from Spain, Chile, and Peru.

Figure 3 aims to summarize, in percentage terms, the composition of avocados from each country. For this purpose, the compounds have been grouped into families (as shown in Table 2), their concentrations have been added up, and the percentage represented by each class (with respect to the total metabolites considered in this study) has been calculated. Succinic acid has not been considered in this representation, as it has a magnitude that would prevent any distribution from being correctly seen in the figures.

The average metabolic profile of the Spanish fruits was characterized by 77% phenolic acids and related compounds, 12% nucleosides and amino acids, 5% flavonoids, 4% vitamins, and 2% phytohormones. The average composition of Chilean avocados did not differ much from that just described, with the following percentages: 67% phenolic acids and related compounds, 22% nucleosides and amino acids, 5% vitamins, 5% phytohormones, and 1% flavonoids. The samples from Chile had (in percentage terms) less phenolic acids and more amino acids and nucleosides and phytohormones than the Spanish avocados. The compositional profiles of the samples from Peru were different from those just described, with the following mean distribution in decreasing order of the percentages: 44% amino acids and nucleosides, 39% phenolic acids and derivatives, 14% vitamins, and 3% phytohormones. The content of epicatechin (flavonoids) was very low and accounted for practically none of the total metabolites determined. Therefore, the compositional pattern of the Peruvian samples was more in balance than the others regarding the phenolic acid and amino acid-nucleoside families’ percentage content. Pantothenic acid also constituted a higher percentage of the total in avocados from Peru than in those from Chile and Spain.

### 2.4. Preliminary Results to Select Potential Markers Linked to the Geographical Origin of Avocado cv. Hass

**Figure 4:**
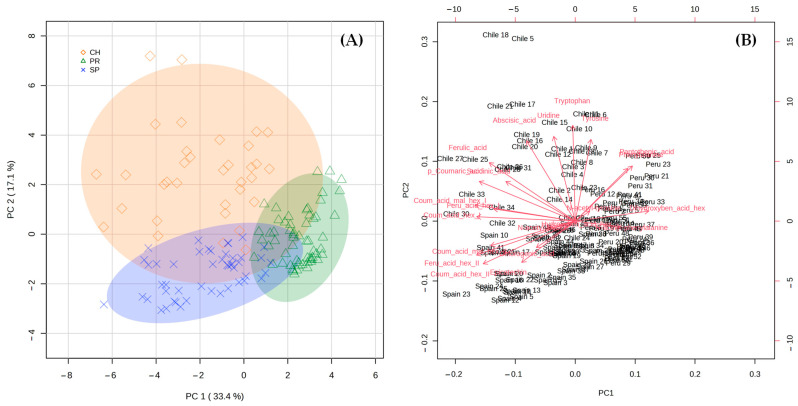
Principal component analysis score plot (**A**) and biplot (**B**) obtained using the LC-MS quantitative data set in a two-dimensional (2D) plot using the first two principal components (2PCs).

PCA was carried out to explore the natural grouping of the samples as well as to point out some possible markers that could be linked to the geographical origin of the avocado fruits. The PCA score plot (Figure 4A) and biplot (Figure 4B) obtained using the LC-MS quantitative data set were displayed in a two-dimensional (2D) plot using the first two principal components, which covered 33.4% (PC1) and 17.1% (PC2) of the total variance, respectively. The accumulated variance explains 71.6%, reaching 5PC’s. Different pre-treatment methods were tested in our data set, i.e., mean centering, auto scaling, pareto scaling, and range scaling, before setting up the best one for this particular case. After several evaluations and the exploration of the nature of the data, auto scaling was the pre-treatment used. The aim of such a strategy is to compare metabolites based on correlation and become all metabolites with equal importance [37].

As seen in the PCA score plot (Figure 4A), Chile was the origin whose samples showed the greatest deviation or heterogeneity between them, whereas those from Peru and Spain were closer to each other in the representation, indicating a greater homogeneity of avocados of these two origins. In addition, it is possible to observe a certain natural grouping of the samples according to the country of origin, suggesting that the metabolite profiling of fruits could enable distinguishing between *Hass* avocados from Peru, Chile, and Spain. 

This work was aimed at describing the typical profiles of avocados from different origins rather than “discriminating” the samples, but using statistical tools to pinpoint the most characteristic features of each origin proved to be very meaningful. Observations in both the PCA and previous sections of this work indicate that some compounds could be useful in defining the distinctive characteristics of the fruits of each origin. By checking the PCA biplot (Figure 4B), it is possible to state that the values of coumaric acid malonyl-hexose II, coumaric acid hexose II, ferulic acid hexose II, epicatechin, and chlorogenic acid were found to be significant in Spanish avocados. Moreover, some phenolic acids and derivatives, as well as uridine, tryptophan, tyrosine, and ABA were important in defining the Chilean avocado’s metabolic pattern versus those of the other origins. Similarly, pantothenic acid, phenylalanine, *N*-acetyl-phenylalanine, and dihydroxybenzoic acid hexose could be considered as markers to identify Peruvian *Hass* avocado fruits. 

**Figure 5:**
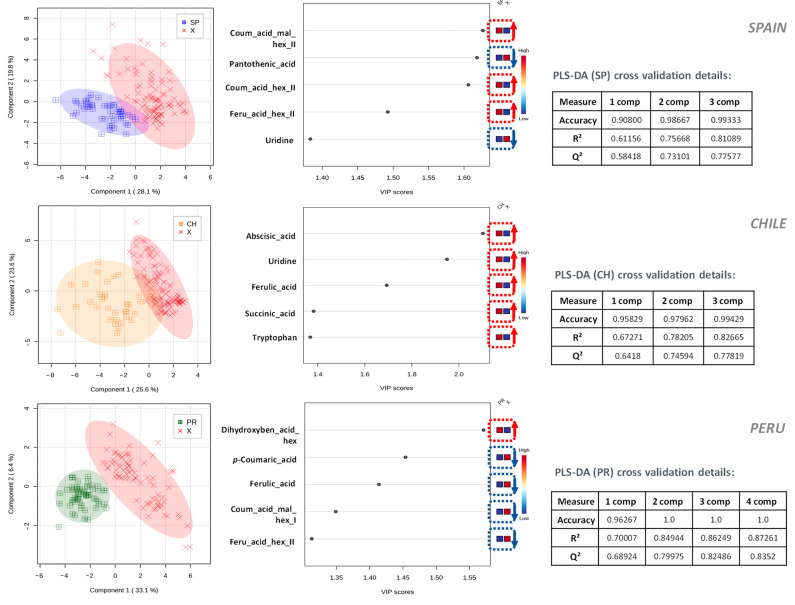
Two-dimensional partial least square analysis score plot (right column), compounds with the highest VIPs in each model (center column), and quality parameters of the model (left column) obtained using the LC-MS quantitative data set. The latter parameters were obtained after cross-validation (accuracy, R^2^, and Q^2^).

To further explore this direction, PLS-DA analysis was used, where a model was built to separate the samples from each country from the rest of the extracts that comprised the sample set. Figure 5 shows the results achieved by applying this strategy, showing from left to right the score plots, the compounds with the highest VIP in each model (together with their relative concentration in each class of the model), and the quality parameters of the model obtained after cross-validation (accuracy, R^2^, and Q^2^).

Thus, the most important features to distinguish avocados from Spain were the following: high levels of coumaric acid malonyl-hexose II, coumaric acid hexose II, and ferulic acid hexose II, together with considerably low levels of pantothenic acid and uridine. Chilean avocado fruits were distinguished from avocados from other countries by their remarkably high concentrations of ABA, uridine, ferulic acid, succinic acid, and tryptophan. As far as avocados from Peru are concerned, it is possible to describe their characteristic pattern as follows: high concentrations of dihydroxybenzoic acid hexose, alongside very low levels of *p*-coumaric acid, ferulic acid, coumaric acid malonyl-hexose I, and ferulic acid hexose II.

## 3. Materials and Methods

### 3.1. Chemicals and Reagents

All reagents were of analytical or LC-MS grade and were used as received in the laboratory. Double deionized water with a conductivity of 18.2 MΩ, obtained using a Milli-Q system (Millipore, Bedford, MA, USA), was used to prepare phase mobile A. Acetic acid, used for the acidification of mobile phase A, was supplied by Panreac (Barcelona, Spain). LC-MS grade acetonitrile (mobile phase B) from Lab-Scan (Dublin, Ireland) was also used. The entire volume of the prepared mobile phase was filtered through a 0.45 µm Nylaflo^TM^ nylon membrane filter, which was supplied by Pall Corporation (Ann Arbor, MI, USA).

Methanol was the solvent selected for the preparation of the working solutions and the metabolite extraction; it was provided by Prolabo (Paris, France). The pure standards used were uridine, succinic acid, phenylalanine, pantothenic acid, tryptophan, chlorogenic acid, epicatechin, *p*-coumaric acid, ferulic acid, and abscisic acid, all of which were supplied by Sigma-Aldrich (St. Louis, MO, USA). *β*-Estradiol, provided by Sigma-Aldrich, was used as an internal standard (IS) to assess the reproducibility of the analytical process. Stock solutions of each analyte were prepared in methanol. Prior to injection, each extract or standard mixture was filtered with a 0.22 μm Clarinet^TM^ nylon syringe filter (purchased from Bonna-Agela Technologies (Wilmington, DE, USA)) and stored in amber vials at −23 °C. 

### 3.2. Samples

Spanish avocado fruits were provided by the Institute for Mediterranean and Subtropical Horticulture (IHSM La Mayora-UMA-CSIC) located in Algarrobo-Costa, Málaga. Approximately, 240–250 fruits of *Hass* avocado from Spain (*n* = 48) were harvested between January and July covering the early, mid, and late *Hass* avocado harvesting seasons in Spain. During this period, samples were taken approximately every 3 weeks, considering a total of 10 different samplings (dates of reception or time points). Spanish avocados’ dry matter (DM) ranged from 25% at the beginning of the season to 32% in the last months of fruit harvesting. All DM measures were performed on unripe fruits according to the AOAC 920.151 method [38]. Avocado fruits from Chile and Peru, covering the periods of time in which there is no domestic *Hass* production in Spain, were provided by the avocado supplier company Trops, which imports avocado from those countries to supply the European market when no *Hass* from Spain is available. Regarding avocados from Peru, about 290–300 fruits (*n* = 59) were received between June and September (every 1–1.5 weeks, collecting a total of 12 sampling points). About 160–170 Chilean avocado fruits were received between October and November (approximately every week for a total of 6 samplings, *n* = 34). DM of Chilean avocados ranged from 23% to 27%, while Peruvian avocados varied from 24% to 30%, as they covered a longer period. Fruits from Chile and Peru were imported in controlled atmosphere by transoceanic transport and were received in optimal conditions. To ensure the representativeness of the sampling, for each geographical origin, each sample was composed of mesocarp aliquots taken from 5 different fruits; in addition, at each sampling point (each date of reception), about 5 samples were taken.

Avocado fruits were ripened under ambient conditions (20–25 °C) until they reached the “ready-to-eat” stage. Ripe avocados were peeled and cut in half to remove the pit. Once the mesocarp was separated, it was cut into strips, sampled, freeze-dried, crushed, homogenized, and frozen at −26 °C. 

A quality control (QC) avocado extract was prepared by mixing an equivalent volume of each extract from all the avocado samples under study and was utilized for instrument control and method validation.

### 3.3. Extraction Procedure

Sample extracts were prepared in duplicate following the solid–liquid extraction protocol described by Serrano-García and collaborators with certain modifications [28]. IS was added before starting the sample preparation to assure the repeatability of the whole analytical protocol. A portion of 0.25 g of freeze-dried sample was extracted twice with 20 mL of pure methanol. Both extraction steps consisted of 2 min of vortex shaking, ultrasound bath for 30 min, and centrifugation (5 min at 9000 rpm). After phase separation, the supernatants were mixed and evaporated, and the residue was redissolved in 1 mL of methanol and filtered using a 0.22 µm pore size nylon syringe filter. 

### 3.4. LC-MS and Statistical Analyses

Qualitative studies were performed using an Acquity UPLC™ H-Class system coupled to a high-resolution (HR) MS detector (QTOF SYNAPT G2 MS (Waters, Manchester, UK)). The analytical platform used for quantitative analyses was a 1260 Infinity Agilent (Agilent Technologies, Waldbronn, Germany) coupled with an Esquire 2000 Ion Trap (IT) low-resolution (LR) mass spectrometer (Bruker Daltonics, Bremen, Germany). Both instruments worked with an electrospray (ESI) interface. Identical chromatographic conditions were applied to both LC systems. The column used was a Zorbax Eclipse Plus C18 column (4.6 × 150 mm, 1.8 μm particle size) and was thermostated at 25 °C. Analytes were eluted with 0.5% acetic acid in water (mobile phase A) and pure acetonitrile (mobile phase B) applying the following gradient: 0 min, 95% A and 5% B; 22 min, 25% A and 75% B; 23 min, 0% A and 100% B; 23.5 min, 0% A and 100%; 25 min, initial conditions. The flow rate was set at 0.8 mL min^−1^, and an injection volume of 10 μL was used both for extracts and pure standards.

Data from both MS analyzers were acquired in full scan mode in a mass range from 50 to 1000 m/z and negative polarity. The ionization source in the IT MS worked under the following conditions: 30 psi for the nebulizer gas (nitrogen), 9 L min^−1^ for dry gas (nitrogen) flow rate, and 300 °C as dry gas temperature. The capillary voltage was set at +3200 V and the end-plate offset at −500 V. For HR MS analyses, all these parameters were transferred and adapted to the ESI-QTOF MS system. The ionization source in the HR MS system operated at +2100 V, 100 °C in the capillary, and 100 L h^−1^ of cone gas flow at 500 °C. The AutoMS data acquisition mode, based on the fragmentation of the prevalent precursor ion per scan, was used to acquire the MS/MS spectra.

MassLynx (Waters), Agilent ChemStation (Agilent Technologies), and Esquire Control (Bruker Daltonics) were the software used for instrument control. DataAnalysis 4.0 software (Bruker Daltonics, Bremen, Germany) was used for data processing, and Microsoft Excel v2204 for data representation. MetaboAnalyst v5.0 was the software applied to carry out supervised and unsupervised statistics (principal components analysis (PCA) and partial least squares-discriminant analyses (PLS-DA)). Auto scaling normalization was selected as a pre-processing step. In the first stage, the natural clustering of the samples was studied by performing a PCA with a data matrix composed of 22 variables (the quantified compounds) and 141 samples (the total sample set comprising the samples from Peru, Chile, and Spain). Subsequently, three different two-class models were built using PLS-DA (one for each geographical origin versus the rest of the samples) to show the characteristic compositional patterns of each producing region. Full cross-validation was performed to evaluate the predictive power of the obtained models. The Hierarchical Clustering Heatmap, used for intuitive visualization of the entire data set, was completed using a Euclidean distance measure and Ward clustering method. 

### 3.5. Analytical Parameters of the Method

Pure standards solutions and QC extracts were used to establish the figures of merit of the applied analytical method. The linear calibration ranges, LODs, LOQs, and repeatability were established for the 10 analytes that were available as pure standards. Solutions of uridine, succinic acid, phenylalanine, pantothenic acid, tryptophan, chlorogenic acid, epicatechin, *p*-coumaric acid, ferulic acid, and ABA were prepared in pure methanol at 10 different concentrations levels. The concentration range for each compound was defined by considering the previously described concentration levels for that substance in avocado mesocarp samples, as well as the results of our preliminary studies with the sample set of this research. Calibration curves were obtained for each standard by least squares regression, each point on the line being the mean value of three separate injections (*n* = 3). In case the pure commercial standard was not available or could not be obtained, the analyte was quantified with another compound of the same chemical family (assuming that they would be expected to exhibit similar responses). Four coumaric acid-derived compounds, hydroxybenzoic acid hexose, and dihydroxybenzoic acid hexose were quantified in terms of the *p*-coumaric acid pure standard. Two ferulic acid derivatives (ferulic acid hexoses) were quantified with its aglycone standard (ferulic acid). Tyrosine, *N*-acetyl-tyrosine, *N*-acetyl-phenylalanine, and *N*-acetyl-leucine were quantified with the phenylalanine pure standard.

LOD and LOQ of each individual compound were estimated by calculating the concentration that generated the signal-to-noise ratio (obtained at the lowest concentration level of those tested) equal to 3 and 10, respectively. Precision was evaluated in terms of repeatability. Intra-day repeatability was expressed as a coefficient of variation (% CV) from 7 injections of the QC extracts performed within the same sequence. Inter-day repeatability was obtained from 18 injections of the same QC extract performed in different sequences and days.

## 4. Conclusions

Since self-sufficiency in avocado consumption in Europe is currently unattainable, imports of avocados from other continents are necessary. In this work, a comprehensive characterization of the metabolic profile of *Hass* avocados marketed in Europe and originating from Spain, Chile, and Peru was carried out by LC-MS. A total of 22 compounds from different categories were determined, their concentrations were compared between samples, and the relative proportions of each of these chemical classes (as a percentage) in the total metabolic profile were established. Finally, PCA and PLS-DA were applied to the data analysis.

The levels of epicatechin, coumaric acid malonyl-hexose II, coumaric acid hexose II, and ferulic acid hexose II were higher in Spanish avocados. Chilean avocados stood out in terms of uridine, tryptophan, ABA, succinic acid, and several phenolic acids content. Peruvian avocados exhibited notable concentrations of *N*-acetyl-phenylalanine, phenylalanine, pantothenic acid, and dihydroxybenzoic acid hexose. Thus, the obtained results, with the help of statistical models, defined avocado fruit compositional patterns for each geographical origin. It might help to obtain comparative nutritional information on the avocados available in the market (domestic or not) throughout the year. 

## Figures and Tables

**Table 1:** Peak assignment of the metabolites found in the *Hass* avocado samples from Spain, Chile, and Peru.

Compound	Family	Molecular Formula	Rt (min)	*m/z* _experim_	*m/z* _theoret_	Error (ppm)	mSigma Value	MS/MS
Uridine	Nucleoside	C_9_H_12_N_2_O_6_	2.7	243.0610	243.0623	0.7	6.9	199.9 [M-H-43]^-^
Tyrosine	Amino acid	C_9_H_11_NO_3_	3.0	180.0662	180.0666	2.5	18.2	162.9 [M-H-17]^-^
Succinic acid	Organic acid	C_4_H_6_O_4_	3.2	117.0192	117.0193	1.0	8.8	-
Dihydroxybenzoic acid hexose	Phenolic acid derivative	C_13_H_16_O_9_	4.9	315.0715	315.0722	2.0	3.6	152.9 [M-H-162]^-^
Phenylalanine	Amino acid	C_9_H_11_NO_2_	4.9	164.0720	164.0717	−1.7	16.6	146.9 [M-H-17]^-^
Unknown 1	-	C_13_H_22_O_10_	5.0	337.1139	337.1140	0.3	12.1	-
Pantothenic acid	Vitamin	C_9_H_17_NO_5_	5.2	218.1028	218.1034	2.7	17.9	145.9 [M-H-28-44]^-^
Hydroxybenzoic acid hexose	Phenolic acid derivative	C_13_H_16_O_8_	6.3	299.0770	299.0772	0.7	52.8	-
Tryptophan	Amino acid	C_11_H_12_N_2_O_2_	6.5	203.0818	203.0826	4.1	2.1	159.0 [M-H-44]^-^ 115.9 [M-H-28-44]^-^
*N*-acetyl-tyrosine	Amino acid derivative	C_11_H_13_NO_4_	6.6	222.0772	222.0772	−0.2	7.3	179.9 [M-H-42]^-^ 162.9 [M-H-42-17]^-^107.0
Chlorogenic acid	Phenolic acid	C_16_H_18_O_9_	7.1	353.0887	353.0878	−2.4	25.1	191.0 [M-H-caffeic moiety]^-^
Coumaric acid hexose I	Phenolic acid derivative	C_15_H_18_O_8_	7.5	325.0934	325.0929	−1.5	6.6	162.9 [M-H-162]^-^ 145.0 [M-H-162-18]^-^
Coumaric acid hexose II	Phenolic acid derivative	C_15_H_18_O_8_	7.8	325.0917	325.0929	3.6	6.5	162.9 [M-H-162]^-^ 145.0 [M-H-162-18]^-^
Ferulic acid hexose I	Phenolic acid derivative	C_16_H_20_O_9_	7.8	355.1033	355.1035	0.3	21.4	192.9 [M-H-162]^-^
Unknown 2	-	C_16_H_22_O_8_	7.8	341.1242	341.1242	0.0	4.8	298.8 [M-H-42]^-^ 280.9 [M-H-162]^-^
Ferulic acid hexose II	Phenolic acid derivative	C_16_H_20_O_9_	8.1	355.1027	355.1035	2.2	22.1	192.9 [M-H-162]^-^
Epicatechin	Flavonoid	C_15_H_14_O_6_	8.3	289.0711	289.0718	2.3	8.5	244.9 [M-H-44]^-^
*N*-acetyl-leucine	Amino acid derivative	C_8_H_15_NO_3_	8.6	172.0974	172.0979	2.8	22.1	-
Coumaric acid malonyl-hexose I	Phenolic acid derivative	C_17_H_20_O_9_	9.0	367.1021	367.1035	3.8	3.4	162.9 [M-H-162-86]^-^ 144.9 [M-H-180-86]^-^
Coumaric acid malonyl-hexose II	Phenolic acid derivative	C_17_H_20_O_9_	9.3	367.1027	367.1035	2.1	14.9	162.9 [M-H-162-86]^-^ 145.0 [M-H-180-86]^-^
*N*-acetyl-phenylalanine	Amino acid derivative	C_11_H_13_NO_3_	9.4	206.0817	206.0823	2.5	27.1	163.9 [M-H-42]^-^ 146.9 [M-H-42-17]^-^
*p*-Coumaric acid	Phenolic acid	C_9_H_8_O_3_	9.9	163.0395	163.0401	1.5	6.2	118.9 [M-H-44]^-^
Ferulic Acid	Phenolic acid	C_10_H_10_O_4_	10.4	193.0498	193.0506	4.3	17.2	177.8 [M-H-15]^-^ 133.9 [M-H-15-44]^-^
Unknown 3	-	C_9_H_16_O_4_	11.4	187.0976	187.0976	0.0	10.9	-
Unknown 4	-	C_14_H_24_O_6_	12.3	287.1492	287.1500	0.1	19.4	227.0 [M-H-60]^-^ 209.0 [M-H-18]^-^
Abscisic acid	Phytohormone	C_15_H_20_O_4_	12.9	263.1286	263.1289	1.2	3.9	219.0 [M-H-44]^-^ 153.0 [M-H-44-66]^-^
Unknown 5	-	C_9_H_16_O_3_	13.6	171.1020	171.1027	4.1	5.4	152.9 [M-H-18]^-^ 127.0 [M-H-44]^-^

When MS/MS information is not included for some compounds, it is because clean fragmentation spectra were not obtained (due to low concentration or difficult cleavage of their labile bonds). Some of the different *m/z* values observed in the MS/MS spectra correspond to typical losses of −17 (NH_3_), −18 (H_2_O), −28 (CO), −42 (C_2_H_2_O), −43 (CHNO), −44 (CO_2_), and −162 (hexose). The prevalent ion detected in the MS spectra of coumaric acid malonyl-hexose I and II was [M-H-44]^-^, corresponding to a *m/z* signal of 367.

**Table 2:** Summary of the quantitative results obtained for the avocado samples obtained from Spain, Chile, and Peru. The values are expressed as mg kg^1^ of dry weight (global SD is indicated for each value).

Chemical Class	Spain		Chile		Peru	
Compound	Mean	SD	Mean	SD	Mean	SD
Amino acids and nucleotides						
*N*-acetyl-leucine	3.35	2.37	1.99	0.42	1.24	0.50
*N*-acetyl-phenylalanine	16.76	9.02	10.24	2.77	19.31	11.84
*N*-acetyl-tyrosine	13.33	2.77	9.96	2.45	24.25	15.84
Phenylalanine	5.42	2.33	11.05	6.30	18.11	7.30
Tryptophan	1.58	0.66	5.98	3.49	2.65	0.94
Tyrosine	2.62	0.76	9.18	5.85	5.74	3.80
Uridine	18.68	6.85	96.72	10.14	41.46	6.71
Flavonoids						
Epicatechin	27.82	17.48	7.11	6.51	0.05	0.03
Organic acids						
Succinic acid	836.34	170.16	1295.68	263.90	383.93	139.43
Phytohormones						
Abscisic acid	11.22	3.87	30.81	7.77	7.89	3.42
Phenolic acids and related compounds						
Chlorogenic acid	1.89	1.49	1.14	0.76	0.55	0.37
Coumaric acid hexose I	165.16	37.33	212.37	30.49	18.67	14.09
Coumaric acid hexose II	49.08	33.63	22.62	18.89	3.99	3.73
Coumaric acid malonyl-hexose I	44.70	18.38	49.52	28.71	8.62	7.21
Coumaric acid malonyl-hexose II	51.54	21.28	26.95	13.58	12.51	10.56
Dihydroxybenzoic acid hexose	6.18	0.94	9.25	4.21	35.96	11.30
Ferulic Acid	8.08	2.46	13.57	3.56	3.06	1.40
Ferulic acid hexose I	10.78	3.20	19.25	18.29	1.74	1.56
Ferulic acid hexose II	6.35	3.59	3.99	3.33	0.37	0.23
Hydroxybenzoic acid hexose	1.94	1.67	3.40	3.10	3.12	2.38
*p*-Coumaric acid	52.54	20.80	75.40	34.52	11.29	10.48
Vitamins						
Pantothenic acid	20.15	4.84	30.26	4.04	35.14	6.13

## Data Availability

Data are contained within this article or supplementary material.

## Supplementary Materials

The following supporting information can be downloaded at: https://www.mdpi.com/article/10.3390/plants12163004/s1, Table S1. Analytical parameters of the LC-MS method used in the present study; Figure S1. Bar diagrams of the twenty-two compounds quantified in *Hass* avocados from Chile, Peru, and Spain.
