# Supplementary material for: Assessing the RP-LC-MS-Based Metabolic Profile of Hass Avocados Marketed in Europe from Different Geographical Origins (Peru, Chile, and Spain) over the Whole Season
Source: Plants (Basel). 2023 Aug 20;12(16):3004. doi: 10.3390/plants12163004 (PMC10458757; doi:10.3390/plants12163004)
Supplement: Supplementary file 1 [file plants-12-03004-s001.zip › plants-2575792-supplementary.pdf]

plants

MDPI

Article

Supplementary Materials

Assessing the RP-LC-MS-Based Metabolic Profile of *Hass* Avocados Marketed in Europe from Different Geographical Origins (Peru, Chile, and Spain) Over the Whole Season

Irene Serrano-García [1], Joel Domínguez-García [1], Elena Hurtado-Fernández [2], José Jorge González-Fernández [3], José Ignacio Hormaza [3], María Gemma Beiro-Valenzuela [1], Romina Monasterio [1,4], Romina Pedreschi [5,6], Lucía Olmo-García [1,]* and Alegría Carrasco-Pancorbo [1]

[1] Department of Analytical Chemistry, Faculty of Sciences, University of Granada, Ave. Fuentenueva S/N, 18071 Granada, Spain; iserrano@ugr.es (I.S.-G.); jdogar99@correo.ugr.es (J.D.-G.); gemabv@ugr.es (M.G.B.-V.); rmonasterio@ugr.es (R.M.); alegriac@ugr.es (A.C.-P.)
[2] Department of Biological and Health Sciences, Faculty of Health Sciences, University of Loyola. Campus Sevilla, Avda. de las Universidades S/N, 41704 Dos Hermanas, Sevilla, Spain; emhurtado@uloyola.es
[3] Institute for Mediterranean and Subtropical Horticulture (IHSM La Mayora-UMA-CSIC), 29750 Algarrobo-Costa, Málaga, Spain; jorgegonzalez-fernandez@eelm.csic.es (J.J.G.-F.); ihormaza@eelm.csic.es (J.I.H.)
[4] Instituto de Biología Agrícola de Mendoza (IBAM), UNCuyo-CONICET, Facultad de Ciencias Agrarias, Chacras de Coria, 5505 Mendoza, Argentina
[5] Pontificia Universidad Católica de Valparaíso, Escuela de Agronomía, Facultad de Ciencias Agronómicas y de los Alimentos, Calle San Francisco S/N, La Palma, 2260000 Quillota, Chile; romina.pedreschi@pucv.cl
[6] Millennium Institute Center for Genome Regulation (CRG), 8331150 Santiago, Chile.
* Correspondence: luciaolmo@ugr.es; Tel.: +34-958-249510

Index

Table S1. Analytical parameters of the LC-MS method used in the present study.

Figure S1. Bar diagrams of the twenty-two compounds quantified in *Hass* avocados from Chile, Peru, and Spain.

Table S1. Analytical parameters of the LC-MS method used in the present study.

Compound	*Rt (min)*	*Calibration curves*	r^2	*LOD (µg L^{-1})*	*LOQ (µg L^{-1})*	*Lineal range (mg L^{-1})*	*Repeatability (% CV)*	
							Intra-day[a]	*Inter-day*[b]
Uridine	2.7	y = 7087.8 + 15744.5x<br>y = 157088.5 + 6364.1x	0.9910<br>0.9945	40.2	133.8	LOQ - 16.1<br>16.1 – 64.3	8.90	9.23
Succinic acid	3.2	y = 1887.9 + 1302.5x<br>y = 29928.5 + 577.6x	0.9939<br>0.9910	118.4	395.4	LOQ – 20.1<br>20.1 – 643.4	7.15	14.63
Phenylalanine	4.9	y = -184.1 + 35428.7x<br>y = 19534.4 + 31628.4x	0.9992<br>0.9983	20.1	67.1	LOQ – 1.7<br>1.7 – 26.8	7.53	12.45
Pantothenic acid	5.2	y = 250.3 + 66086.8x<br>y = 28703.0 + 54982.3	0.9984<br>0.9968	35.4	118.0	LOQ – 0.8<br>0.8 – 26.8	9.25	10.08
Tryptophan	6.4	y = 1557.6 + 58178.7x<br>y = -3198.1 + 62650.8x	0.9973<br>0.9997	22.5	75.0	LOQ – 0.8<br>0.8 – 13.4	7.85	10.34
Chlorogenic acid	7.1	y = -152.6 + 28634.7x<br>y = 11441.8 + 22052.8x	0.9962<br>0.9956	62.1	206.7	LOQ – 1.3<br>1.3 – 10.7	8.26	10.83
Epicatechin	8.3	y = 21437.4 + 65579.4x<br>y = 894997.8 + 25710.0x	0.9905<br>0.9944	13.6	45.2	LOQ – 10.7<br>10.7 – 85.8	7.73	10.38
p-Coumaric acid	9.9	y = 87289.2 + 23255.4x<br>y = 1837252.8 + 6750.0x	0.9983<br>0.9911	17.8	59.1	LOQ – 90.5<br>90.5 – 723.9	7.21	9.51
Ferulic acid	10.4	y = 5700.8 + 37765.4x<br>y = 124533.4 + 15519.3x	0.9928<br>0.9954	13.8	46.1	LOQ – 5.4<br>5.4 – 21.5	9.30	10.30
Abscisic acid	12.9	y = 7005.9 + 125830.9x<br>y = 366668.8 + 57711.5x	0.9988<br>0.9909	7.1	23.6	LOQ – 4.0<br>4.0 – 32.2	6.76	7.83

Abbreviations used: Rt (Retention time); LOD (Limit of detection); LOQ (Limit of quantification).
a: coefficient of variation (%) corresponding to injections (n = 7) of the QC sample performed in the same sequence.
b: coefficient of variation (%) corresponding to injections (n = 18) of the QC sample carried out in sequences carried out on different days.

Phenolic acids and related compounds

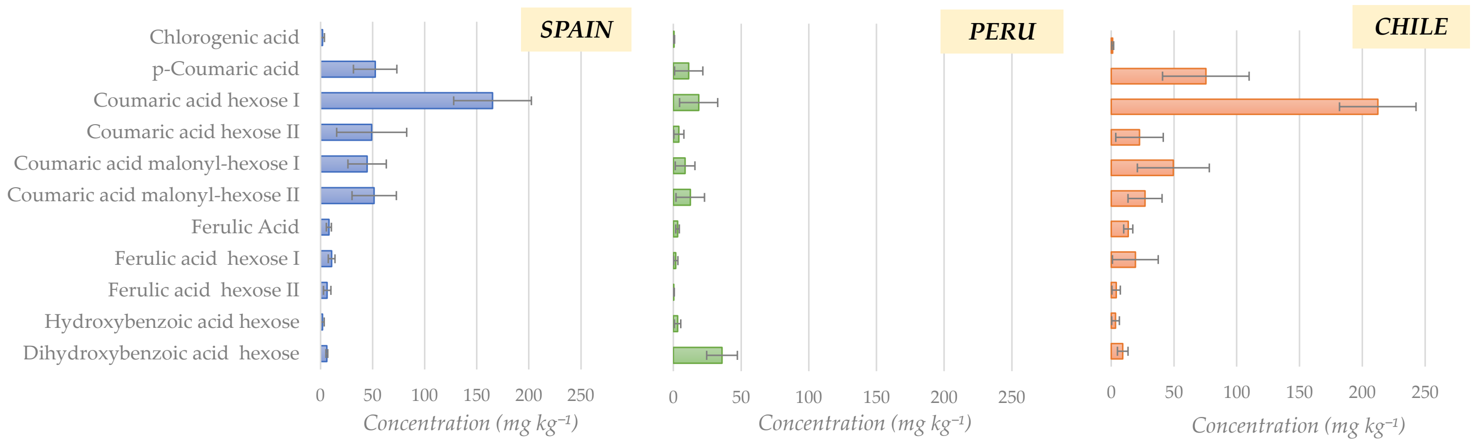

Amino acids and nucleosides

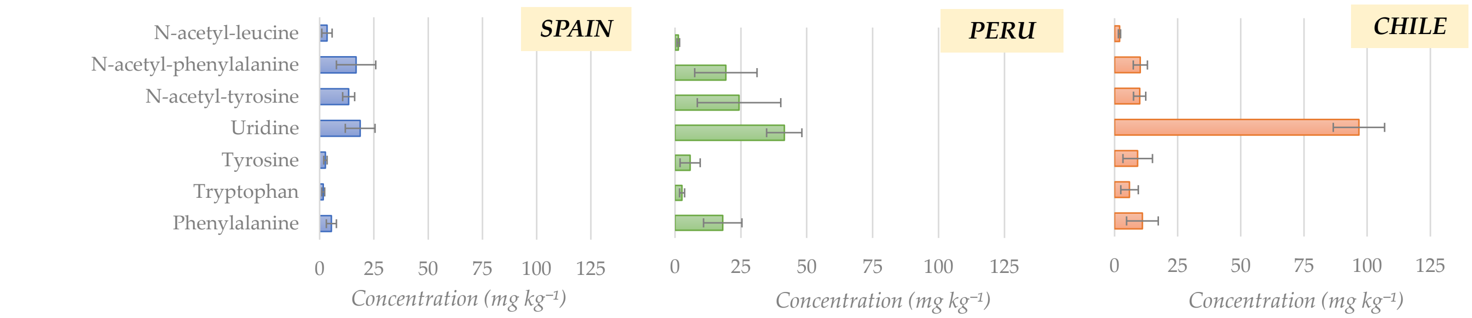

Other compounds (vitamins, flavonoids and phytohormones)

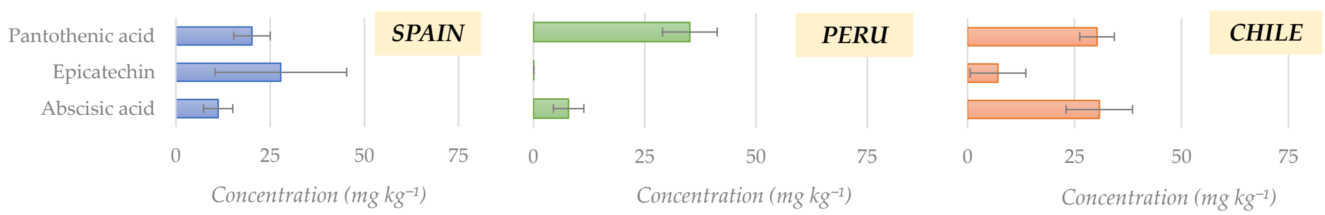

Succinic acid (organic acid)

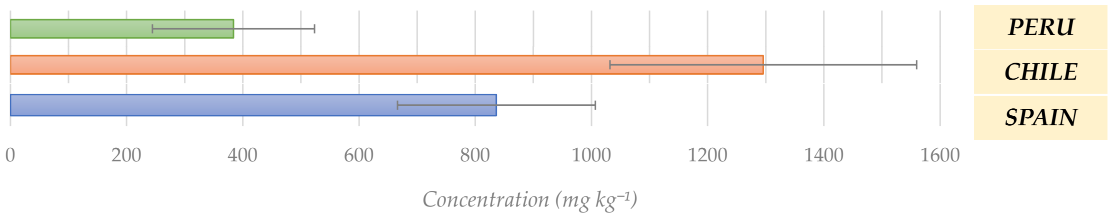

Figure S1. Bar diagrams of the twenty-two compounds quantified in *Hass* avocados from Chile, Peru, and Spain. Compounds are grouped by chemical families and results are expressed as the mean of each geographical origin in mg kg^{-1} with associated standard deviations (SD).
